# ISCB Honors 2021 Award Recipients Peer Bork, Barbara Engelhardt, Ben Raphael, Teresa Attwood

**DOI:** 10.1093/bioinformatics/btab383

**Published:** 2021-07-12

**Authors:** Christiana Fogg, Diane Kovats, Martin Vingron

**Affiliations:** Freelance Writer, Kensington, MD 20895, USA; International Society for Computational Biology, Leesburg, VA 20176, USA; Max Planck Institute for Molecular Genetics, Berlin 80539, Germany

## Abstract

Annually, the International Society for Computational Biology (ISCB) recognizes three outstanding researchers for significant scientific contributions to the field of bioinformatics and computational biology, as well as one individual for exemplary service to the field. ISCB is honored to announce the 2021 Accomplishments by a Senior Scientist Awardee, Overton Prize recipient, Innovator Awardee and Outstanding Contributions to ISCB Awardee.

Peer Bork, EMBL Heidelberg, is the winner of the Accomplishments by a Senior Scientist Award. Barbara Engelhardt, Princeton University, is the Overton Prize winner. Ben Raphael, Princeton University, is the winner of the ISCB Innovator Award. Teresa Attwood, Manchester University, has been selected as the winner of the Outstanding Contributions to ISCB Award.

Martin Vingron, Chair, ISCB Awards Committee noted, ‘As chair of the Awards Committee it gives me great pleasure to convey my heart-felt congratulations to this year’s awardees. Our community, as represented by the committee, admires these individuals’ outstanding achievements in research, training, and outreach.’

## 2021 ISCB Accomplishments by a Senior Scientist Award: Peer Bork

International Society for Computational Biology (ISCB) recognizes a leader in the fields of computational biology or bioinformatics annually with the Accomplishments by a Senior Scientist Award. This is the highest award bestowed by ISCB in recognition of a scientist’s significant research, education and service contributions. Peer Bork, Director of EMBL Heidelberg (Scientific Activities), is being recognized with the 2021 Accomplishment by a Senior Scientist Award. He will receive his award and present a keynote address at the 2021 Joint Intelligent Systems for Molecular Biology (ISMB)/European Conference on Computational Biology (ECCB) being held virtually on July 25–30, 2021.

Peer Bork grew up in Berlin, East Germany before the fall of the Berlin Wall. Bork was interested in math as a young student and his math teacher encouraged him to join the chess club and compete in Math Olympiad, for which he eventually competed at a national level. Bork was most interested in logic because he enjoyed deducing mathematical concepts, and his early interest in math helped him to gain entry into a specialized math school for 9th–12th class. He was also a consummate reader and devoured books that explained biological phenomena like photosynthesis. Bork considered studying science as a viable career path, as he recalled, ‘I was always a curious person that used analytical thinking, and having grown up in East Germany, science was an area where I hoped that I could stick to facts with limited impact of political propaganda.’ He attended the specialized math school in the early 1980s, where he was first exposed computer programming through training on a Russian computer that was nearly the size of a car. Soon after, PCs were available in East Germany, and Bork had his first experience using computation to solve a biological problem as a young graduate student at the University of Leipzig. He said, ‘In a practicum during my biochemistry studies, I successfully optimized the commercial production of certain NADH-dependent dehydrogenases in a fermenter by computationally simulating reaction equations.’ This experience helped him develop a project for his computational diploma (equivalent to a master’s degree) work in 1987, in which he worked to understand the evolution of enzyme cofactor binding domains in the context of substrate binding and turnover. He recalled, ‘For this I had to collect sequences of those enzymes, search emerging sequence databases, align them using early multiple alignment tools, and extend my mini-dataset by doing homology searches using a self-designed and self-coded (with a colleague) sequence pattern approach. The respective research field around sequence analysis was still small, but the databases expanded rapidly, and it was enjoyable to get to biological novelty quickly by using homology inferences (e.g. binding site prediction), identifying novel protein domains or broadening existing ones using sequence signatures. It was not uncommon to get results sufficient for a good scientific paper within a week or so, and as a byproduct, one could learn a lot about molecular biology while reading the papers around the published protein sequences. I was fascinated by the independent evolution of such domains, and I shifted focus from enzymatic domains to more mobile ones to decipher the modular LEGO principle of building blocks for protein function. I stored the domains I discovered and/or described in a self-made database. This was rewarding and efficient, and I soon had enough material to be able to finish my PhD in 1.5 years. With this experience, I was already an expert and pioneer in a quickly expanding research field with a high impact on biological research.’ Bork’s PhD experience has shaped his selection of research topics, as he prefers to tackle nascent research areas for which little is known, and he takes a more data-driven rather than hypothesis-driven approach, thus freeing him of lofty expectations of experimental outcomes.

**Peer Bork: From Behind the Wall to the Microbiome**


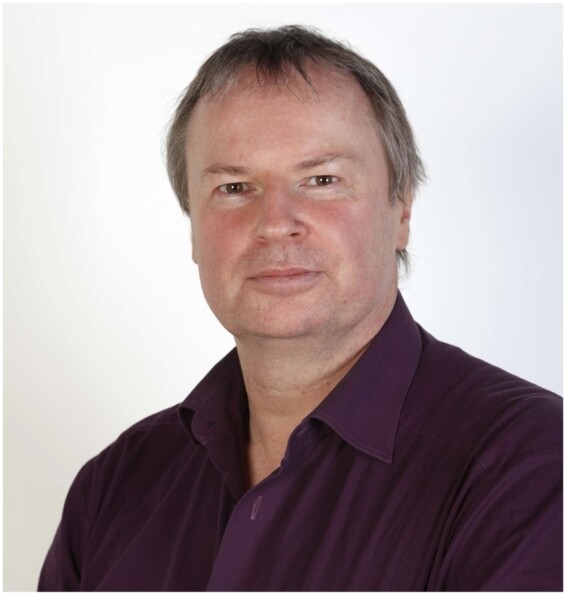


Bork credits much of his development into an independent researcher to his mentorship under his PhD supervisor Jens Reich (Berlin). He recounted, ‘When I joined his group in 1988, he was one of the leaders of the political underground movement in East Germany, shortly before the wall came down. He was under constant surveillance by the “Stasi,” and it was unclear whether he would end up in prison. He never made a fuss about his political standing, and at work he encouraged and helped me without micromanagement. A couple of years later, he was a runner-up for the German Federal Presidency. He treated everybody equally, was a knowledgeable and engaging supervisor and supported me, even during unification, when everyone at my institute lost their jobs and had to re-apply for the few new positions.’ Reich continued to mentor Bork and other students despite his political duties as a member of the German Parliament and other pursuits, including writing essays for newspapers and authoring non-scientific books. Bork also valued the academic guidance that grew out of his work with Russell Doolittle, a world-renowned evolutionary biologist and biochemist at the University of California, San Diego, and said, ‘[Doolittle’s] enthusiasm, wisdom and focus on important things (for him), as well as his use of storytelling in science made a deep impression on me and others, and positively influenced my social skills and devotion to science.’

Bork completed his Habilitation in 1995 at Humboldt University in Berlin. He had concurrently joined EMBL Heidelberg in 1991, became Group Leader there in 1995, was promoted to Head of a unit (equivalent to department) in 2001, served additionally as Strategic Head of Bioinformatics since 2011 and is director of the Heidelberg site since 2020. Bork has made many meaningful contributions to bioinformatics, particularly through his early work on protein domains (SMART database), genome analysis of higher eukaryotes, work on methods for analysis of mutation data (PolyPhen) and large-scale phylogeny (iToL), as well as inventing several methods for inferring gene/protein networks (STRING database) and analysis of drugs and adverse reactions (STITCH and SIDER), and his recent pioneering microbiome research. Bork continues to be fascinated with this field, and said, ‘I moved research subfields a few times but got stuck on microbiomes for more than 15 years, mostly focusing on the human gut microbiome. I witnessed the very beginning of a quickly expanding field and have seen it progress to medical applications. I believe that the progress of the gut microbiome research towards improving human health can be paralleled by global microbiomics towards planetary health and this is a fascinating thought. It seems possible to get a census and understanding of molecular and cellular functions and their evolution for our entire planet. This might enable a much better evolutionary understanding of functions and help in the development of applications for biotechnology, but also towards solving societal questions like antibiotic resistance and general sustainability. I’m also fascinated by the ongoing scientific revolution in structural biology towards high resolution structures of entire cells, enabled by cryoEM technology. This will be yet another qualitatively new baseline understanding with plenty of practical applications.’

Like many scientists, Bork has had his share of unexpected findings that have changed his perspective or research approach. Bork explained, ‘I found our discovery of enterotypes, that is the stratification of the human population into microbial community types surprising, as we could not really explain it, and I think, despite tons of hypotheses, still nobody can really explain this observation. Other examples against my own expectation were phenotypic patterns that were strong enough to pinpoint molecular mechanisms, for example medical drug side effects we could use for drug target identification, or predictions of which human-targeted drugs also impact the gut microbiome. Those findings did not change research strategies though, just encouraged me to remain open-minded in pursuing research.’ He has published over 600 manuscripts, many of which are highly cited, and several of his web resources have stood the test of time, including, SMART, STRING, eggnog, SIDER and iToL, due to an international network of researchers maintaining and further developing these, often led by group alumni.

Bork has supported the bioinformatics community in other capacities, including serving as a Senior Editor of *Molecular Systems Biology* and an Editorial Advisory Board Member of *PLOS Computational Biology* and a reviewer for interdisciplinary journals including *Science* and *Cell*. He has organized numerous EMBO courses and has remained dedicated to nurturing young scientists, for which he was recognized with the 2008 *Nature* award for mentoring in science. His own training under Reich has shaped him into a mentor that encourages students to explore different fields, with a focus on contextualizing these findings with other observations. Bork considers it important to share in strategic aspects of projects and encourages students to chart their own paths and present their work to the greater scientific community.

Bork has built his scientific career on exploring different subfields and methods, and he greatly appreciates his recognition with the 2021 Accomplishment by a Senior Scientist Award as it is conferred by his bioinformatics peers for his many varied contributions to the field.

## 2021 ISCB Innovator Award: Ben Raphael

The International Society for Computational Biology (ISCB) Innovator Award honors an ISCB scientist who is within two decades of his or her graduate degree completion and has consistently made outstanding contributions to the field of computational biology. The 2021 winner is Dr. Ben Raphael, Professor of Computer Science at Princeton University. He will receive his award and present a keynote address at the 2021 Joint Intelligent Systems for Molecular Biology (ISMB)/European Conference on Computational Biology (ECCB) being held virtually on July 25–30, 2021.

**Ben Raphael: From Space Nut to Cancer Conqueror**


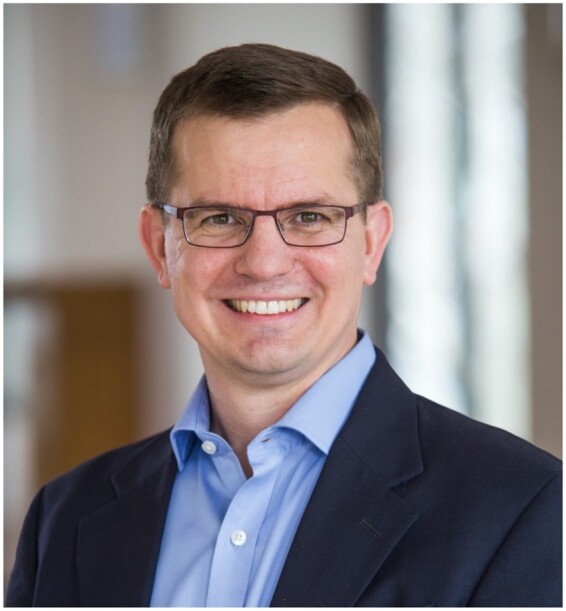


Ben Raphael grew up in the Washington, DC area and was fascinated with science from a young age. His mother was a science teacher and nurtured a love of science in Raphael and his siblings. Raphael was first bitten by the ‘space bug,’ and he fondly recalls his two oldest brothers pooling their money to buy a telescope, which allowed Raphael and his family to enjoy many nights stargazing from their own yard. Raphael immersed himself in books about the space program, watched every shuttle launch, and frequented the nearby National Air and Space Museum. Eventually, Raphael was admitted to the Massachusetts Institute of Technology (MIT), where he pursued a major in mathematics and a minor in biology. Throughout his undergraduate coursework, he started to prefer mathematics courses that used abstract thinking and shifted away from fact-heavy biology courses. Raphael went on to pursue his PhD in Mathematics at the University of California, San Diego (UCSD) under the mentorship of Jim Agler. During his graduate studies, UCSD launched its Bioinformatics Graduate Program, and Raphael enrolled in a bioinformatics course offered by Pavel Pevzner, who had recently joined the UCSD faculty. This course was a major turning point for Raphael, as he recalled, ‘I found computational biology to be an amazing blend of the disciplines that I had pursued: mathematics, computer science and biology. I was enamored by the huge potential of genome sequencing, as the human genome sequence had just been published and the mouse genome was well underway. Moreover, computational biology was such a broad discipline that I thought I would never be bored and could change my focus from mathematical and computational questions to biological questions depending on where my research led me.’

Raphael is deeply appreciative of the mentorship he had early in his career. His PhD advisor Jim Agler not only taught him a great deal about mathematics, but also about not allowing one’s ego or preconceived ideas to hamper the pursuit of truth. Raphael pursued his post-doctoral studies under Pevzner, who introduced him to computational biology and shaped how he approaches research questions to this very day. Raphael was introduced to the field of cancer genomics through his work with Colin Collins and Joe Gray during his post-doc, and these collaborators provided him with valuable guidance and support during his transition to an independent investigator. As a new assistant professor at Brown University, Raphael also received invaluable support from Rick Wilson and Elaine Mardis, who got him involved in The Cancer Genome Atlas (TGCA) project and other large-scale cancer projects. Raphael is now a tenured professor at Princeton, and he relishes the newfound freedom to pursue longer-term projects, while balancing the needs of his students and trainees, who are also building their publication records. Raphael tries to apply lessons learned from his post-doc training under Pevzner, and he said, ‘My training strongly influences how I train my students to select research questions and conduct research. Biology is a vast discipline, and there are a wide range of problems where computational biologists can contribute. I learned from my post-doctoral advisor Pavel Pevzner the importance of clearly formulating a biological problem as a computational problem. In some cases, this formulation reduces the problem to one that is already solved—and perhaps solved with existing software. While there are many problems in biology that can benefit from the application of computational methods, we strive to find problems where there is a need for a new algorithm.’ Raphael is also strongly influenced by his mathematical training, which taught him that writing clear and precise problem statements and definitions can greatly clarify one’s thinking about a complicated problem. He similarly trains his students to write a rigorous statement of the computational problem they are trying to solve, and to define their terminology carefully.

Raphael’s scientific curiosity has been focused for several years on cancer genomics. He continues to be amazed by the mutational heterogeneity observed in different cancers, particularly the long tail phenomenon, for which only a few genes are frequently mutated in cohorts of cancer patients whereas a large portion of genes are rarely mutated. Raphael and his team developed methods to study mutation combinations in different pathways and networks that identified groups of genes that were more frequently mutated. He noticed that this method worked better for some cancers, like glioblastoma but did not work consistently across cancer types, and he believes these differences may be due to limitations of current sequencing methods and available data for different tumor types. Raphael considers single cell DNA sequencing technology as a powerful tool for improving how we visualize the complexities of cancer, but he also sees much work to be done in understanding how tumors develop and change in response to treatment. Advances in single cell and spatial sequencing technologies as well as extensions of these methods to measure multiple parameters in parallel are providing more detailed insights into cancer cell biology and tumor heterogeneity. Raphael also sees the benefits of applying CRISPR technology to biological models, which has enabled analysis of interactions between somatic mutations and CRISPR gene knockouts in cancer cell lines that were otherwise undetected in tumor specimens. In 2020, Raphael also came to appreciate the power of these technologies for studying the immune system in the context of SARS-CoV-2 infection and COVID-19, and how these studies can be applied to vaccine development.

Raphael has been recognized for his research contributions throughout his career, including a Sloan Postdoctoral Fellowship (2002–2004), a Burroughs Wellcome Fund CAREER award at the Scientific Interface (2005), a Sloan Research Fellowship (2010) and an NSF CAREER award (2011–2017). He is considered a leader in algorithmic computational cancer biology research and his work has been published in top-tier scientific and computational biology journals. Raphael has developed several widely used algorithms that include THetA and AncesTree algorithms for analyzing mixtures of cancer cells, Dendrix and Multi-Dendrix algorithms for analyzing mutually exclusive mutations, and the HotNet algorithm for network analysis of cancer mutations.

Raphael has served the greater computational biology community in many ways, including working on the steering committees for the RECOMB Satellite Workshop on Computational Cancer Biology (2007–present) and the RECOMB Satellite Workshop on Massively Parallel Sequencing (2012–present). He has served on the program committees for ISMB, RECOMB, PSB and many other conferences and has helped to organize research programs at UCLA, Bertinoro, the Simons Institute for the Theory of Computing and other venues. Raphael has also reviewed grant proposals for NSF and NIH for most years since 2008. He is a key contributor to The Cancer Genome Atlas (TCGA) and International Cancer Genome Consortium (ICGC) projects and has taken on leadership roles in these projects.

Raphael is honored, humbled and grateful for recognition with the 2021 ISCB Innovator Award. As an ISCB Fellow, he participated in the selection of new Fellows this year and came to appreciate the number of exceptional computational biology researchers being considered for recognition as a Fellow or for an ISCB award. Raphael is deeply grateful for his students and post-doctoral fellows who have worked hard and contributed to the success of many research projects. He is also thankful for the unwavering support of his wife and children through the many phases of his career.

## 2021 ISCB Overton Prize: Barbara Engelhardt

Each year the International Society for Computational Biology (ISCB) recognizes the achievements of an early to mid-career scientist with the Overton Prize. This prize honors the untimely death of Dr. G. Christian Overton, a respected computational biologist and founding ISCB Board member. The Overton Prize honors independent investigators who are in the early to middle phases of their careers and have made significant contributions to computational biology through research, teaching and service.

ISCB is pleased to recognize Dr. Barbara Engelhardt, Associate Professor of Computer Science at Princeton University, as the 2021 winner of the Overton Prize. She will receive her award and present a keynote address at the 2021 Joint Intelligent Systems for Molecular Biology (ISMB)/European Conference on Computational Biology (ECCB) being held virtually on July 25–30, 2021.

**Barbara Engelhardt: A Winding Road that Leads to Statistics**


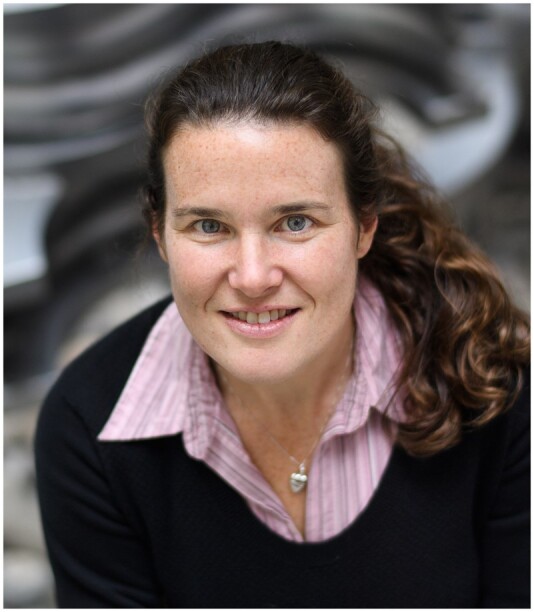


Engelhardt grew up in New York City and recalled that her early encounters with math were rather frustrating. She had been placed in a remedial track in math until her fifth-grade teacher, Ms. Dorian, told her, ‘If I can’t read your work, I can’t grade you well.’ From that moment, Engelhardt was able to express her mathematical prowess more clearly and excelled in upper-level mathematics classes through high school, including recognition with two coveted math awards. As a freshman at Stanford University, Engelhardt enrolled in the challenging honors math series, but she was less than enchanted with the heavy focus on theoretical math. She also took her first computer science class and recalled, ‘Everything clicked. It was less about proving things in math and more about logic and reasoning with math.’ Engelhardt’s next mind-opening experience occurred when she took a machine learning class taught by Prof Daphne Koller, a new hire to the Computer Science department, that brought together many topics that interested her, including Bayesian networks and linear regression. Engelhardt went on to TA for Koller and complete an MS in Computer Science under her mentorship. She then took a position at the Jet Propulsion Laboratory (JPL) for two years, just as many of her peers were getting recruited to Google, which was a little-known startup at the time. Without a PhD, Engelhardt learned she would have limited opportunities for advancement at JPL, so she applied to grad school and landed at the University of California, Berkeley. She trained under Prof Michael Jordan, who not only guided her on her academic pursuits but taught her valuable lessons in mentorship and how to see ‘both the forest and the trees.’ Engelhardt fondly recalls the famous group meetings that Jordan organized with his lab during which they would select a topic to learn about for several weeks and spend hours thinking through machine learning ideas and statistics concepts at great depth, even if they ultimately abandoned a concept they had considered applying to a research project. During grad school, Engelhardt immersed herself in statistics courses and came to really appreciate this area of mathematics because of the ability of statistical methods to explore data, find patterns and solve important problems. She sought out a PhD project focused on a biological problem and collaborated with Prof Steven Brenner, ultimately leading to the completion of her dissertation on predicting protein molecular function in 2007. During her time at Berkeley, she also enjoyed interacting with Prof Monty Slatkin’s group, from which she learned of the fascinating connections between population genetics and statistics. Engelhardt then had a brief stint at the nascent 23andMe and went on to be a post-doc at the University of Chicago under the advisement of Prof Matthew Stephens. She recalled, ‘[Stephens] constantly challenged me and the other people he mentored to understand our research from as many perspectives as possible to be able to best explain and teach the ideas to others, but also because he is a genuinely curious person.’ During her post-doc, she performed research on new statistical approaches for association mapping for complex phenotypes and to identify population structure in genotype data. She considers her post-doc training as the time when she really learned how to think as a Bayesian statistician. Her foundational experiences as a grad student and post-doc honed her perspective on data science: The scientific question is the goal, and the methods used for the analysis of data should be as simple as possible while supporting the scientific goal as directly as possible.

Engelhardt launched her career as an independent researcher in 2011 when she took a position as a visiting research scientist at Duke University’s Biostatistics and Bioinformatics Department and was then hired as an assistant professor. She put her training to work as she sorted through what works and what does not work in Bayesian statistics. During this period, Engelhardt applied statistical models to complex phenotype association studies, differential gene expression analysis and RNA sequencing (RNA-seq) data to better understand mechanisms of human disease. In 2014, Engelhardt joined the Computer Science Department at Princeton University, and she received tenure there in 2018. She has built a research program focused on developing statistical models and machine learning methods to analyze biomedical data, with a focus on identifying and characterizing complex associations, sequential decision making and predicting the effects of perturbations in single-cell data, human cohorts, and medical record data. With her group, she has developed many valuable methods for analyzing and understanding single-cell genomic data, including a scalable and robust approach to dimension reduction using a Gaussian process latent variable model (GPLVM) with t-distributed residuals. Engelhardt has also led several large scale multi-disciplinary collaborations, including making foundational contributions to the Genotype-Tissue Expression (GTEx) Consortium and working with the Hospitals of the University of Pennsylvania to develop models for real-time hospital patient data, including vital signs and lab results, that can forecast these values several days in advance. Throughout her research, she has sometimes come upon negative results that lead her projects in different directions, including a recent observation that she could not really detect differences in outcome between black and white hospitalized COVID-19 patients but did find a five-year average age gap between these cohorts. She has come to value negative findings in a different way and said, ‘But instead of tossing the work, I think “Well, that’s interesting. I wonder why our results don’t match common knowledge in this domain. What does this negative result mean?” This thought process has led my group to some of our best work. These stories take a long time to play out sometimes, much to the frustration of the students working on them, but they are often worth the investment. Also, sometimes these follow up questions don’t require new tailor-made models and methods to find the source of the result; instead, asking for feedback from other scientists and being clever about how to test alternative hypotheses is the most insightful way forward. My favorite research directions are ones in which every dangling string is pulled—whether for methods development, biological questions, or hypothesis testing.’ Engelhardt has expanded her research scope dramatically as an established PI, which she attributes to having students who are interested in different biological problems. She said, ‘The wonderful thing about working in statistics and machine learning is that it is possible to work on problem domains that you haven’t tackled before by finding the right collaborator who is patient with your learning process throughout the collaboration. This means that I’ve been able to take advantage of students’ eagerness to work in medical data records, sociology, neuroscience, bioengineering and psychology because we have found great collaborators and the students to drive those relationships.’

Engelhardt has been recognized for her rigorous and creative statistical approaches with several awards and recognitions, including an NSF CAREER Award and Sloan Faculty fellowship. As a PI, she most treasures her work in mentoring students and post-docs, and she has adapted the positive experiences she had as a trainee to her mentoring approach, including treating each person as a unique individual and fostering independence as well as collaboration. Outside of the lab, she has served the computational biology and greater biomedical communities in several ways, including service as the Diversity & Inclusion Co-Chair for the International Conference on Machine Learning, being a member of NIH Advisory Committee to the Director Working Group on Artificial Intelligence, working as a co-organizer for numerous workshops and meetings, and serving as an associate editor for the Annals of Applied Statistics.

Engelhardt feels tremendously honored to be selected for the Overton Prize, especially since she looks to so many of the previous Overton Prize winners. She also acknowledges that the work she has been recognized for would not be possible without the difficult and creative work carried out by her students and post-docs.

## 2021 ISCB Outstanding Contributions to ISCB Award: Teresa Attwood

The Outstanding Contributions to the International Society for Computational Biology (ISCB) Award was initiated in 2015 to recognize members who have made beneficial and lasting contributions to the Society through their leadership, service and educational work, or a combination of these three areas. Teresa (Terri) Attwood, Professor emerita at the School of Computer Science, The University of Manchester is the 2021 Outstanding Contributions to ISCB winner. She will receive her award at the 2021 Joint Intelligent Systems for Molecular Biology (ISMB)/European Conference on Computational Biology (ECCB) being held virtually on July 25–30, 2021.

Attwood has spent much of her career as a champion of the bioinformatics education community. After completing her PhD in Biophysics at the University of Leeds in just two years, she was awarded a prestigious Royal Society University Research Fellowship (1993–2002) and was also a visiting fellow at the European Bioinformatics Institute (EMBL-EBI). In 2001, she rose to Chair of Bioinformatics at the School of Computer Science at the University of Manchester. Attwood recognized the critical importance of bioinformatics education for the greater scientific community and coauthored one of the first bioinformatics textbooks with David Parry-Smith, as well as two other educational texts coauthored with Paul Higgs, and with Steve Pettifer and Dave Thorne.

Attwood is an esteemed ISCB member who has advocated for bioinformatics education through her years of service with ISCB and the greater bioinformatics and computational biology communities. Attwood first became aware of ISCB when she attended the 1997 ISMB meeting in Halkidiki, and she soon became a regular ISMB attendee, as it was one of the few meetings focused on bioinformatics. She was especially interested in incorporating bioinformatics education into ISMB and contributed to the one-day satellite meeting called the Workshops on Education in Bioinformatics (WEB) that launched in 2001. She continued her bioinformatics education and training advocacy by serving as a member of the ISCB Education Committee and as an ISCB Board Member (2013–2016). During her service as a Board member, Attwood was also Chair of GOBLET (Global Organization for Bioinformatics Learning, Education and Training) and worked closely with Fran Lewitter to better coordinate the objectives and activities of these two organizations to meet the surging worldwide demand for bioinformatics training. They succeeded in incorporating bioinformatics education and training into the ISMB program through the formation of the Education Community of Special Interest (COSI) and brought bioinformatics tutorials to other conferences through the support of ISCB. Attwood acknowledges that Lewitter’s shared passion for bioinformatics education and her desire to work together harmoniously were essential to their shared success in advancing their bioinformatics and training initiatives.

**Teresa Attwood: A Voice for Bioinformatics Education**


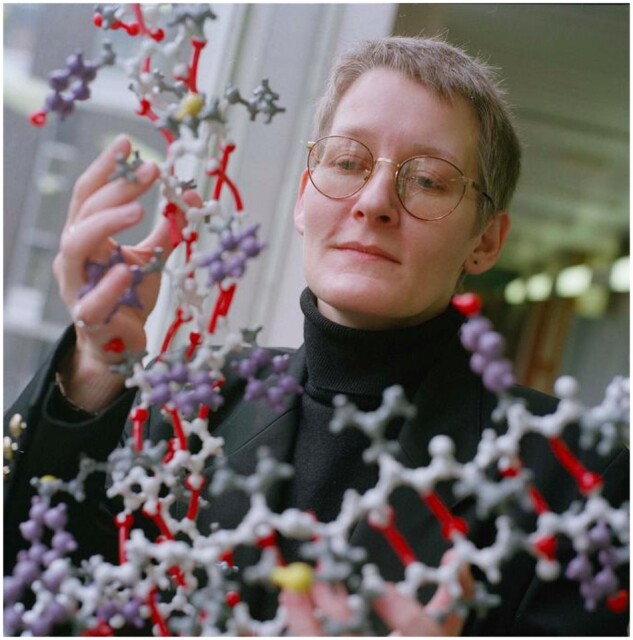


Attwood is recently retired from the University of Manchester and has deeply appreciated her ISCB involvement. She said, ‘I valued my membership in ISCB for creating my first ‘bioinformatics home’, bringing opportunities to meet like-minded people and to become part of a community. These early experiences provided the springboard for embracing a hugely diverse worldwide community, learning about their bioinformatics training needs, their challenges and their innovative solutions. Those first steps paved the way for working with colleagues to create a variety of bioinformatics training materials; to create the GOBLET Foundation and establish the Education COSI; to build the foundations of the bioinformatics training strategy of ELIXIR (Europe’s data infrastructure), and to contribute to the development of its training portal (TeSS) and its Train-the-Trainer program; and to become involved with many other bioinformatics training programmes and initiatives worldwide. Ultimately, these opportunities opened doors to work with high school teachers, and to create materials and resources to help plug gaps in their bioinformatics training needs. It has been immensely gratifying to be able to work with, and to learn from, such experienced, supportive and cherished colleagues across these varied educational contexts. Engaging with them to steer training projects within international societies and foundations (ISCB, ISB, SEB, EMBnet, ELIXIR, GOBLET and H3ABioNet to name but a few) taught me a lot.’

Attwood encourages junior scientists and trainees to seek out service opportunities both to broaden their horizons and to build their networks. She appreciates how involvement in professional societies like ISCB gave her valuable opportunities to serve on working groups, task forces, and committees, and urges other young scientists to get involved in areas that ‘stir their hearts.’ Attwood has deeply valued the experiences that arose from her service to ISCB, but she has most treasured the decades-long relationships that have been built on trust and mutual respect and continue to be of great benefit even in retirement.

## Funding

Funding was provided by the ISCB to C.F. for this commissioned piece.

*Conflict of Interest*: none declared.

